# Serum biomarkers of papillary thyroid cancer

**DOI:** 10.1186/1916-0216-42-16

**Published:** 2013-02-07

**Authors:** Fawaz M Makki, S Mark Taylor, Ali Shahnavaz, Andrew Leslie, Jeffrey Gallant, Susan Douglas, Evelyn Teh, Jonathan Trites, Martin Bullock, Karen Inglis, Devanand M Pinto, Robert D Hart

**Affiliations:** 1 Department of Surgery, Division of Otolaryngology, Queen Elizabeth II Health Sciences Centre and Dalhousie University, 1278 Tower Rd., B3H 2Y9, Halifax, N. S., Canada; 2 Department of Pathology, Queen Elizabeth II Health Sciences Centre and Dalhousie University, 5788 University Ave., B3H 1V8, Halifax, N. S., Canada; 3 National Research Council of Canada, Institute for Marine Biosciences, 1411 Oxford St., B3H 4H7, Halifax, N. S., Canada; 4 QE II Health Sciences Centre, 5788 Tower Rd., B3H 2Y9, Halifax, N. S., Canada

**Keywords:** Thyroid, Cancer, Papillary, Serum biomarkers

## Abstract

**Objective:**

To identify serum biomarkers of papillary thyroid cancer.

**Methods:**

Prospective analysis was performed of banked tumor and serum specimens from 99 patients with thyroid masses. Enzyme-linked immunosorbent assay (ELISA) was employed to measure levels of five serum proteins previously demonstrated to be up-regulated in papillary thyroid cancer (PTC): angiopoietin-1 (Ang-1), cytokeratin 19 (CK-19), tissue inhibitor of metalloproteinase-1 (TIMP-1), chitinase 3 like-1 (YKL-40), and galectin-3 (GAL-3). Serum levels were compared between patients with PTC and those with benign tumors.

**Results:**

A total of 99 patients were enrolled in the study (27 men, 72 women), with a median age of 54 years. Forty-three patients had PTC and 58 cases were benign tumors. There were no statistically significant differences when comparing all five different biomarkers between PTC and other benign thyroid tumors. The p-values were 0.94, 0.48, 0.72, 0.48, and 0.90 for YKL-40, Gal-3, CK19, TIMP-1, and Ang-1, respectively.

**Conclusion:**

Serum levels of four of the five proteins were elevated in patients with thyroid masses relative to normal values. However, the difference between benign and PTC was not significant. Two of the markers (Gal-3 & TIMP-1) displayed a greater potential difference, which may warrant further investigation. This study suggests that other serum markers should be sought. This is the first study to investigate potential serum biomarkers based on over-expressed proteins in thyroid cancer versus benign pathology.

## Introduction

Papillary thyroid cancer (PTC) is one of the few cancers whose incidence is on the rise. It is considered the most rapidly increasing of all cancers in Canada [1]. Incidence has increased by 6.8% per year in males and 8.8% per year in females since 1998 in Canada with similar results noted in Europe and the United States [1]. Risk factors for thyroid cancers include prior radiation, family history and genetics. Treatment by surgery and radio-iodine therapy is highly effective; however, due to the difficulty in accurate diagnosis of thyroid cancer, surgery is often performed on benign nodules. Benign thyroid masses such goiter, follicular adenomas and cysts are approximately ten times more common than thyroid cancers [2]. Pre-operative diagnostic tests such as U/S, CT scan, and fine needle aspirate (FNA) often do not provide a definitive diagnosis. In these indeterminate cases, diagnostic hemithyroidectomy surgery is often performed and up to 75% of these patients have benign disease [3].

Research into the use of genomic techniques for improved diagnosis has focused on transcript profiling of well-differentiated thyroid cancer [4]. Genes that appear to be up-regulated in these studies are numerous. Jazab et al. demonstrated in a group of 16 patients with PTC increased gene expression for lectin, galactoside-binding, soluble, 3 (LGALS3) which codes for galectin-3 (Gal-3) protein, tissue inhibitor of metalloproteinases 1 (TIMP1), and chitinase 3-like 1 (CHI3L1) which is also known as YKL-40 [5]. Baris et al. noted high expression of adenosine deaminase (ADA), eukaryotic translation initiation factor 2 subunit 2 (EIF2S2), TIMP1, cyclin D1 (CCND1), cadherin-3 (CDH3) and collagen, type VIII, alpha 1 (COL8A1) in PTC [6]. Niedźwiecki et al. explored the role of angiopoietin 1 and 2 in thyroid cancers, showing a negative correlation of angiopoietin-1 (Ang-1) with state of malignancy [7]. Based on a review of these and other published studies using gene expression analysis, we concluded that YKL-40, Gal-3, cytokeratin 19 (CK19), TIMP-1 and Ang-1 were consistently found to be upregulated.

An alternative approach to tumor transcript profiling is protein profiling of serum. The main advantages of this approach are that it is minimally invasive, relatively safe and can be performed repeatedly. This could provide the basis for a useful test for screening, diagnosis, treatment and followup. However, the high complexity of serum makes identification of biomarkers challenging. Relative to healthy controls, the levels of serum vascular endothelial growth factor (VEGF) and matrix metallopeptidase 9 (MMP-9) are elevated in late-stage PTC; however, as the levels of these markers do not differentiate between goiter and early stage PTC, they are not useful for improving diagnosis [8]. Levels of biotinidase, clusterin, cysteine-rich, angiogenic inducer, 61 (CYR61), enolase 1, nucleolin and prothymosin alpha (PTMA) have been compared between PTC patients and healthy controls but no comparison was made with patients with benign nodules [9]. In spite of these advances, there are currently no validated serum markers for the diagnosis of PTC or for risk stratification of thyroid masses.

Our objective was to identify potential serum markers in subjects with PTC. We hypothesized that markers that have been shown to be highly expressed in PTC tumor tissue, especially those that are secreted or present at the cell surface, would also be elevated in serum, and thus measurable by serum enzyme-linked immunosorbent assay (ELISA) analysis.

## Materials & methods

### Subjects

This was a prospective study. All patients provided informed consent and the study was approved by the local Hospital Ethics Committee. All patients ≥ 18 years old and undergoing hemi-thyroidectomy or total thyroidectomy from July 2009 to June 2011 were enrolled initially in the study. Patients with human immunodeficiency virus or viral hepatitis were not included in the study for investigator handling safety reasons. After post-operative pathological evaluation, all patients with thyroid cancer other than PTC were excluded. Patients were then divided into two main groups based on their diagnosis: 1) PTC group and 2) control group. The control group included all patients with benign thyroid pathology. All specimens were then bar-coded and patient identifiers removed in order to protect their anonymity and also to blind the operators conducting the ELISA analysis.

### Specimen collections (serum & tissue)

In the operating room prior to induction of general anesthesia, 2 red top vials of blood were drawn from all patients. The tubes were left at room temperature for 20-30 minutes to clot and then centrifuged at 3000 rpm for 10 minutes. If serum separation from erythrocytes was incomplete, the centrifugation step was repeated. The serum was aliquoted into 5-8 300 μl microcentrifuge tubes in order to limit the number of freeze-thaw cycles to one. At the same time, immediately upon completion of the procedure, the thyroid specimen was processed by a single study pathologist (M.B.). A representative portion of the mass was also preserved in ribonucleic acid stabilizing reagent (RNAlater, Ambion) for future analysis. Both serum and tissue specimens were subsequently stored in a freezer at -80°C.

### Enzyme-linked immunosorbent assay

All the collected serum specimens were analyzed by ELISA according to the manufacturers’ instructions. ELISA kits for Ang-1 (catalog number DANG10), TIMP-1 (catalog number DTM100) and Gal-3 (catalog number DGAL30) were from the Research and Diagnostics (R&D) Systems, Inc., Minneapolis, MN, USA. The kit for YKL-40 (catalog number Microvue YKL-40 8020 (QI)) was made by Quidel Corp., San Diego, CA, USA. The CK19 ELISA kit (catalog number CSB-E09180H) was from CUSABIO, (http://www.cusabio.com). All ELISA washing steps were performed using a BioTek Elx50 microplate strip washer programmed as required. All readings were made on a Spectramax 384 Plus plate reader (Molecular Devices, Sunnyvale, CA, USA) using the SoftMax Pro software package. All samples, standards and blanks were analyzed in duplicate.

### Statistical analysis

P values for the comparison of the means of the PTC and control groups for each of the 5 serum proteins were determined using t-tests. Random forest classification was performed using the R package random Forest. The random forest analysis was carried out with parameters *ntree*, *mtry* and *nodesize* set to 50 000, 3 and 1 respectively. Normal values for each of the 5 serum proteins were taken from references as indicated [10-14]. 

## Results

**Figure 1:**
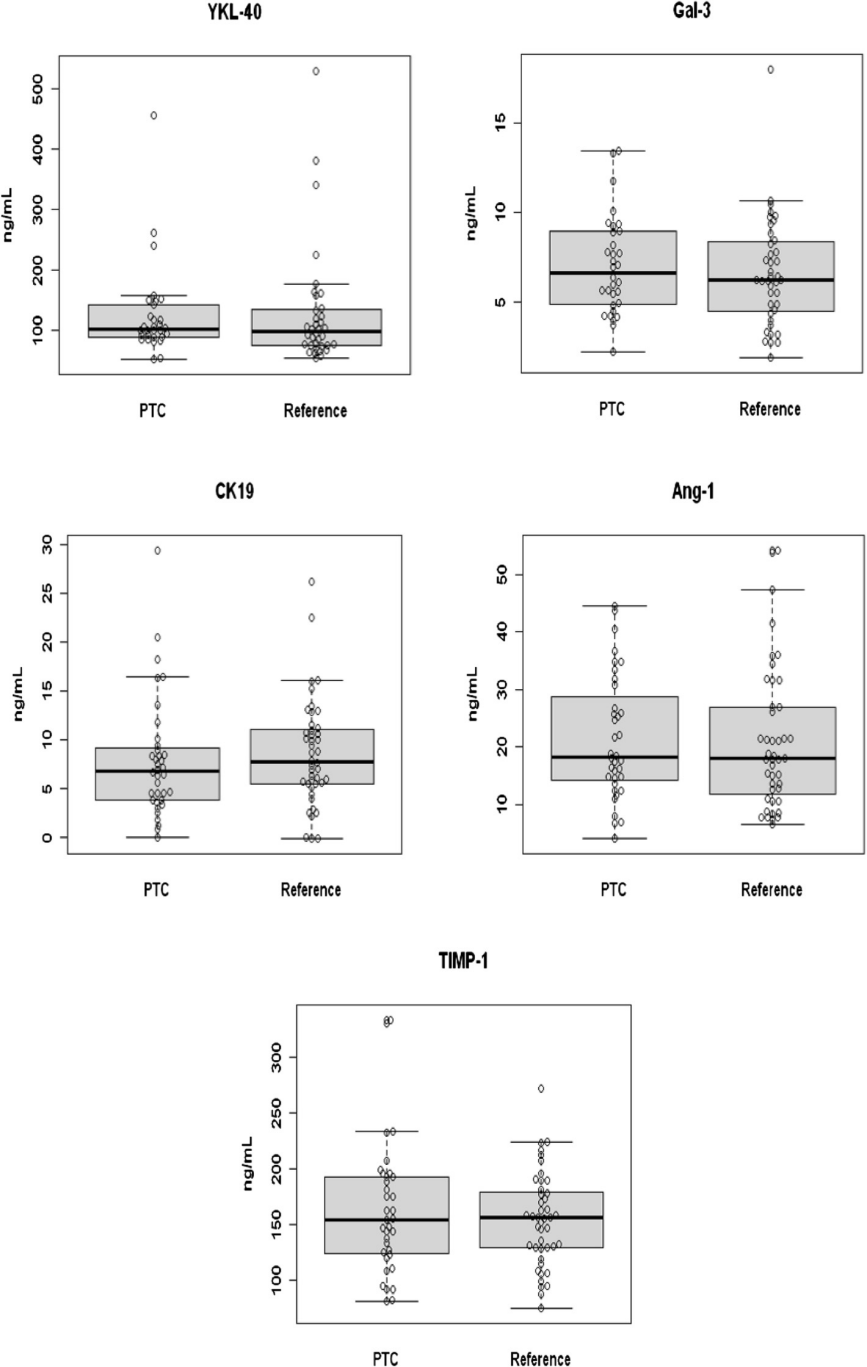
**Boxplot of serum marker levels in the PTC and reference group, which consists of all benign conditions.** Boxes contain 25th and 75th percentile.

**Figure 2:**
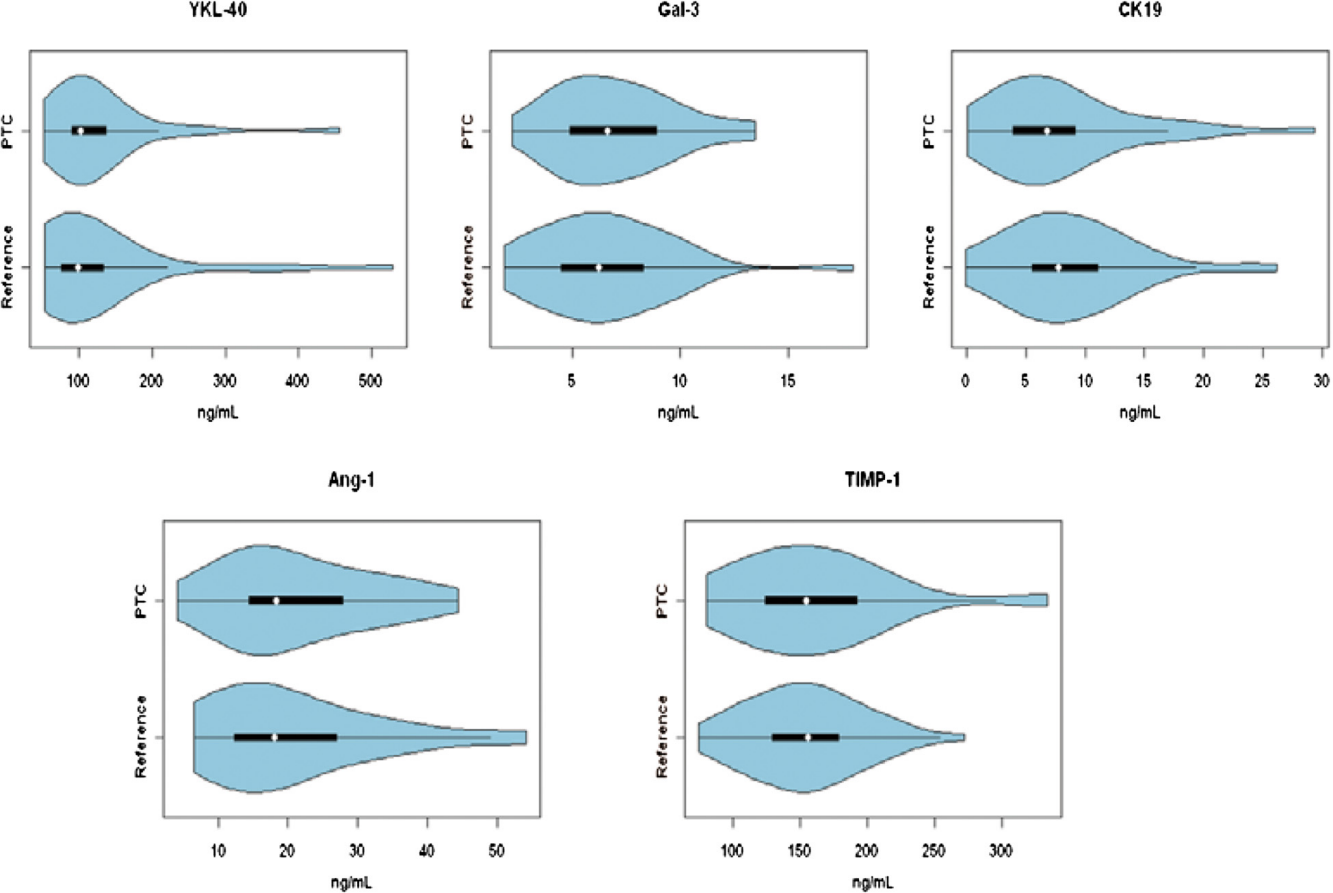
**Violin plot of serum marker levels in the PTC and reference group, which consists of all benign conditions.** Width of the object is proportional to the number of patients with a particular serum concentration.

A total of 99 patients were enrolled in the study (42 PTC & 57 benign). The patients’ characteristics are summarized in Tables 1 &2. Each of the five potential serum markers were measured by ELISA and the levels were compared between PTC patients and the control group, consisting of all benign thyroid conditions. As can be seen in Table 3 & Figure 1, no significant differences were detected for any of the proteins measured. Therefore, the serum levels of these proteins are not diagnostic using univariate statistical analysis. The serum levels of these markers were also plotted using violin plots (Figure 2). In these plots the width of the image is proportional to the number of patients at each serum value. This can aid in the identification of smaller subsets of patients with elevated levels in cases where the mean is dominated by the bulk of the patient values. However, again in this case, no significant sub-groups are apparent.

**Table 1:** Patients characteristics

	**Age**				**Sex**		
	***Min***	***Max***	***Mean***	***Median***	***Male***	***Female***	***Total***
**PTC**	19	78	52.9	54	17	25	42
**Benign**	30	81	53.7	54	10	47	57
**All**	19	81	53.4	54	27	72	99

**Table 2:** PTC patients’ and tumor characteristics

***#***	***Sex***	***Age***	Dx*	***Procedure***	***Level 6 (yes = 1, no = 0)***	***Level 6 nodes (positive/total)***	***Lateral neck (yes = 1, no = 0)***	***Neck nodes (positive/total)***	***Tumor diameter (cm)***	LVI* (yes = 1, no = 0)	ETE* (yes = 1, no = 0)	***Multicentricity (yes = 1, no = 0)***	***Number of foci***
***1***	M	42	MP*	Hemi *(Lt)	1	0/3	0	_	0.1	0	0	0	1
***2***	F	53	PTC*	Total Thyroid*	1	1/12	0	_	0.7	0	1	0	1
***3***	F	38	MP	Hemi (Rt)	0	0	0	_	0.3	0	0	1	2
***4***	F	69	PTC	Hemi (Lt)	1	6/16	1	4/98	0.2	0	0	1	3
***5***	M	59	MP	Total Thyroid	1	0/17	0	_	0.2	0	0	0	1
***6***	M	55	PTC	Total Thyroid	1	0/7	0	_	0.3	0	0	1	5
***7***	F	35	PTC	Total Thyroid	1	0/18	0	_	5.5	0	0	1	7
***8***	F	42	MP	Hemi (Rt)	0	0	0	_	0.2	0	0	1	2
***9***	F	36	PTC	Hemi (Rt)	0	0	0	_	3	0	0	1	6
***10***	M	46	PTC	Hemi (Lt)	0	0	0	_	6.5	1	0	1	2
***11***	F	19	PTC	Total Thyroid	1	3/4	1	10/37	7	1	1	1	+*
***12***	M	63	PTC	Total Thyroid	1	0/7	0	-	1	0	0	0	1
***13***	M	57	PTC	Total Thyroid	1	4/13	0	-	2	1	1	0	1
***14***	M	67	PTC	Total Thyroid	1	0/2	0	_	1.3	0	0	1	4
***15***	M	40	PTC	Total Thyroid	1	11/15	1	13/44	0.5	1	0	1	10
***16***	M	66	MP	Hemi (Lt)	1	0/1	0	_	2.5	0	0	0	1
***17***	F	53	PTC	Total Thyroid	1	0/5	0	_	4	0	0	1	2
***18***	M	77	PTC	Total Thyroid	0	0	0	_	0.3	0	0	1	3
***19***	F	56	PTC	Hemi (Lt)	0	0	0	_	1.7	0	0	0	1
***20***	F	47	PTC	Hemi (Rt)	0	0	0	_	1.1	0	0	1	3
***21***	F	58	PTC	Total Thyroid	1	0/6	0	_	1.6	0	0	1	2
***22***	F	56	PTC	Total Thyroid	1	0/20	0	_	2	1	1	1	2
***23***	M	53	PTC	Total Thyroid	1	7/18	1	11/46	1.8	0	1	1	+
***24***	F	75	PTC	Total Thyroid	1	0/13	0	_	0.5	0	0	1	2
***25***	F	35	PTC	Hemi (Lt)	0	0	0	_	1.3	0	0	1	3
***26***	F	41	PTC	Total Thyroid	1	3/12	0	_	1.7	0	0	1	10
***27***	F	62	PTC	Total Thyroid	1	0/13	0	_	2.2	0	0	0	1
***28***	M	42	PTC	Total Thyroid	1	4/6	0	_	1.7	1	1	0	1
***29***	F	72	PTC	Hemi (Rt)	0	0	0	_	1.5	0	0	0	1
***30***	M	37	PTC	Total Thyroid	1	0/11	0	_	4.5	0	0	0	1
***31***	M	72	PTC	Total Thyroid	1	7/9	1	9/18	2.5	1	1	1	+
***32***	F	37	PTC	Total Thyroid	1	0/9	0	_	1.5	0	0	0	1
***33***	F	40	PTC	Total Thyroid	1	0/6	0	_	0.9	0	0	1	4
***34***	F	40	PTC	Total Thyroid	1	7/8	0	_	4	1	0	1	2
***35***	F	35	PTC	Total Thyroid	1	1/13			1.o		0	0	1
***36***	M	78	PTC	Hemi (Rt)	0	0	0	_	8	0	0	0	1
***37***	F	61	MP	Hemi (Rt)	0	0	0	_	<0.1	0	0	0	1
***38***	M	77	MP	Total Thyroid	0	0	0	_	0.4	0	0	0	1
***39***	F	70	MP	Hemi (Rt)	0	0	0	_	0.2	0	0	1	2
***40***	M	56	PTC	Total Thyroid	1	2/3	1	0	2.5	1	1	0	1
***41***	F	49	MP	Hemi (Lt)	0	0	0	_	0.1	0	0	0	1
***42***	F	56	MP	Hemi (Rt)	0	0	0	_	0.1	0	0	0	1

* *Dx*: Diagnosis, *ETE*: Extra thyroid extension, *Hemi*: Hemithyroidectomy, *Lt*: Left, *LVI*: Lymphovascular Invasion, *MP*: Micropapillary, *PTC*: Papillary Thyroid Cancer, *Rt*: Right, Total thyroid: Total thyroidectomy, +: Innumerable.

**Table 3:** Tumor marker serum measurement values

	**YKL-40**	**Gal-3**	**CK-19**	**Ang-1**	**TIMP-1**
**PTC**	129 ± 77	7.07 ± 2.79	8.1 ± 6.1	21 ± 11	162 ± 60
**Reference**	129 ± 99	6.6 ± 3.0	8.6 ± 5.4	21 ± 12	154 ± 42
**P-value**	0.995	0.488	0.730	0.483	0.910
**Normal**^**a**^	43 ± 25 [10]	2.2 [11]	1.4 +0.3 [12]	58 ± 5 [13]	95 + 26 [14]

* All concentrations in ng/ml, ^a^ Values taken from references as indicated.

**Figure 3:**
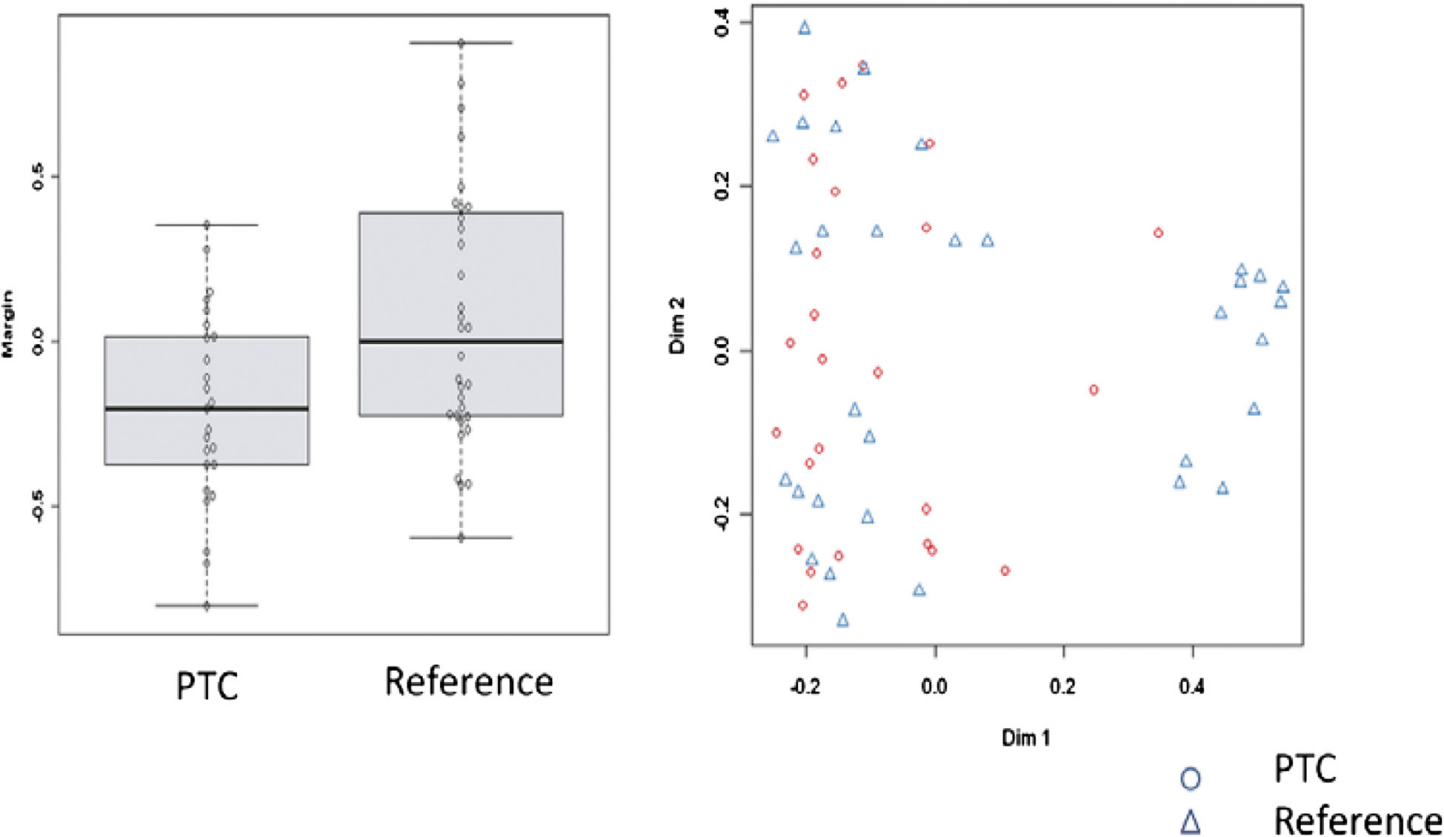
**Boxplot of the margin of prediction (positive values indicate correct classification).** Multi-dimensional scaling plot of the random forest proximity measure.

The potential markers were also analyzed using multivariate statistical analysis. Specifically, the data were analyzed using random forest analysis using R [15]. In this technique, approximately two thirds of the samples were chosen at random to generate a classification tree. At each tree node a random subset of variables (*mtry*) was selected with the variable responsible for the largest split used to split the node. This process was iterated *ntree* number of times to create an ensemble of classification trees in which sample prediction is accomplished through a majority vote. As in the case of the univariate analysis, the multivariate analysis using random forest did not reveal any serum markers that were significant for diagnosis. In Figure 3, the box plot shows that the margin, which is a measure of the ability to distinguish the two groups using the serum marker levels, is close to zero. This indicates poor predictive ability. Similarly, the plot on the right of Figure 3 shows little separation for the PTC and reference groups.

## Discussion

Up to this date the gold standard for the diagnosis of thyroid cancer remains pathological evaluation of surgically obtained thyroid tissue. Since the introduction of FNA in the 1970s the preoperative diagnostic accuracy of thyroid nodules has greatly improved [16]. However, FNA still has significant limitation in the diagnosis of thyroid nodules with an overall sensitivity and specificity from 65-98% and 72-100%, respectively [17]. Microarray technology has significantly improved the understanding of cancer pathophysiology as well by providing valuable clinical information. This has yielded multiple genes that have been demonstrated to be up-regulated in PTC. Certain genes encode secretory proteins that with the use of commercially available ELISA kits are measurable in serum. In 2001, Huang et al. published the first study describing genes that are up-regulated in PTC based on microarray [18]. Comparing 8 pairs of normal and PTC thyroid tissue resulted in the detection of 24 genes that were up-regulated in PTC [18]. In the present study we measured serum levels of YKL-40, Gal-3, CK19, TIMP-1 and Ang-1 proteins using a commercially available ELISA kit.

### Tissue inhibitor of metalloproteinase-1 (TIMP-1)

As its name suggests, TIMP-1 inhibits a variety of metalloproteinases. Its expression is dependent on BRAF and NF-κB using *in vitro* models, where it also influences proliferation [19]. High expression of TIMP-1 is indicative of an invasive phenotype. In 2000, Maeta et al. investigated the expression of both the TIMPs and matrix metalloproteinases (MMPs) in PTC, which were up-regulated relative to normal tissue [20]. Similar results were also obtained by others and confirmed at the protein level [21-25]. Serum levels in thyroid cancer patients have not been previously measured; however, levels in pancreatic cancer patients and, to a lower extent in chronic pancreatitis, have been shown to have diagnostic potential [26]. In metastatic breast cancer patients, elevated serum TIMP-1 levels were shown to correlate with poor response to therapy and lower overall survival [27]. In our study the TIMP-1 levels in PTC and control samples were 162 ng/ml and 154 ng/ml, respectively. The serum level did not show any significant difference despite the strong evidence in the literature for elevated gene and protein expression of TIMP-1. This is the first study to demonstrate equivalent levels of serum TIMP-1 in patients with PTC and benign thyroid nodules.

### Chitinase 3-like 1 (YKL-40)

Chitiniase 3-like 1 has been found to be up-regulated in PTC [18] and in numerous other cancers where it appears to promote angiogenesis [28]. The serum levels of this protein, which is also known as YKL-40, have not been studied in PTC patients. However, it has been found to be elevated in the serum of patients suffering from various other cancers although it is also elevated in various inflammatory conditions [29]. The mean concentration of YKL-40 in both PTC and control samples was 129 ng/mL, which is higher than the level reported in healthy controls (43 ng/ml) [10].

### Lectin, galactoside-binding, soluble, 3 (LGALS3), galectin – 3 (Gal-3)

Galectins are a family of carbohydrate-binding proteins with a high affinity for β-galactoside. Gal-3 is implicated in cell growth and differentiation, cell adhesion, angiogenesis, tumor progression, apoptosis and metastasis and has been detected in many human tumors and cell lines, including thyroid, colon and breast. It has been identified in a number of different tumors, and Xu et al. reported that it is expressed preferentially in papillary and follicular carcinoma at a high level and in medullary carcinoma at a variable level [30]. Bartolazzi et al. used monoclonal antibodies to Gal-3 on 1009 archival tissues and 226 fresh specimens and reported 99% sensitivity and 98% specificity in discriminating benign from malignant lesions [31]. However, a recent study on PTC has observed that there was a decreased intensity of Gal-3 expression at the invasive edges of the tumors during tumor progression [32]. In 2010 Htwe et al. showed a significant (p = 0.001) stronger expression of Gal-3 in the advancing follicular cells at the periphery of thyroid cancer lesions, which was more pronounced in PTC than follicular carcinoma of the thyroid [33]. In our study the mean value of Gal-3 in PTC and control samples were 7.07 and 6.6 ng/ml, respectively, and the p-value was 0.48. These results are consistent with those of Saussez et al. who observed elevated Gal-3 levels in patients with PTC but found significant overlap with Gal-3 levels in patients with benign conditions [11].

### Cytokeratin 19 (CK19)

Cytokeratins are filamentous proteins that participate in the integrity and cytoskeleton of the cells. CK19 has the lowest molecular weight of all the cytokeratins. It is normally expressed in ductal epithelium (bile ducts, pancreas, and renal collecting tubules) and in the mucosa of the gastrointestinal tract. The differential expression of cytokeratins has been evaluated in various thyroid lesions. Among various cytokeratins, CK19 has shown diffuse and strong cytoplasmic staining in PTC, which makes it useful in the diagnosis of PTC [34-39]. High molecular weight cytokeratin has also been found to be significantly increased in PTC and has been reported to be helpful in distinguishing it from other benign and malignant thyroid nodules [37,40]. In 2007, Park et al. had showed that CK19 immunoreactivity of follicular adenoma was higher than previously reported cases, and because of its low specificity this may limit its utility as a diagnostic marker [41]. Our serum protein measurement didn’t show any significant difference between PTC and benign thyroid pathology.

### Angiopoietin-1 (Ang-1)

Angiopoietins (Ang1 and Ang2) are structurally related growth factors, and contribute to angiogenesis upon binding to Tie2 receptor [42]. Ang1 is known as a Tie2 agonist capable of inducing EC migration and proliferation [43-46]. Interaction of angiopoietins with vascular endothelial growth factors (VEGF) in different kinds of tumor tissue is important for tumor growth and possible local invasion or distant metastasis [47-49]. In 2006, Niedźwiceki et al. measured serum levels of Ang-1 using ELISA and showed statistically significant lower concentrations of Ang-1 in patients with thyroid cancers versus patients without thyroid pathology [7]. In contrast, in 2011, Hsueh et al. demonstrated the expression of Ang-1, Ang-2, and Tie-2 in normal benign and PTC tissues and significantly high expression of Ang-1 in metastatic PTC cases [50]. Based on their results they also concluded that Ang-1, Ang-2, Tei-2 receptor, and VEGF play important roles in progression of clinical stages of PTC [50]. Our measurements were consistent with those of Niedźwiceki et al. [7] levels of Ang-1 were lower in PTC compared to normal. However, there was no difference between serum Ang-1 levels in PTC and the benign nodule group.

Despite significant evidence in the literature for elevation of these markers in tumor tissue, the same is not true for serum. Underlying theories why serum values of these markers were not consistent with the tissue values are unknown at this stage. In tumor tissue, the availability of adjacent normal tissue facilitates identification of markers. No such internal control is possible for serum analysis; therefore, in the case of serum markers, there must be a very large difference in concentration in order for the marker to be useful for diagnostic purposes.

## Conclusion

Although the results of this study did not demonstrate a statistically significant difference in serum levels of YKL-40, Gal-3, CK19, TIMP-1 and Ang-1 between PTC patients and those with benign conditions, it does demonstrate the potential feasibility of a blood-based diagnostic test. It also demonstrates that although there may be up-regulation of several of these gene products in PTC tissue, this may not be representative of overexpression of the proteins in serum.

With the rising incidence of thyroid cancer the need for an accurate diagnostic test is becoming paramount and development of such a test may significantly reduce patient morbidity and unnecessary surgery in benign thyroid disease. Obviously further study is required.

## Competing interests

The authors declare that they have no competing interests.

## Authors’ contributions

FM, AS, & KI: Ethics approval, patient enrollment, blood and tissue collection and serum extraction. SD selected candidates for ELISA and assays were performed by JG at the National Research Council. SMT & JT: staff head & neck surgeons. MB: pathologist. DP, AL, & TE: data acquisition and tracking. All authors read and approved the final manuscript.

## References

[B1] Chappell H Pritwish D Dryer D Ellison L Logan H MacIntyre M Canadian cancer Society’s steering committee on cancer statistics 2011 Toronto, ON: Canadian Cancer Society

[B2] Meinhold CL Ron E Schonfeld SJ Alexander BH Freedman DM Linet MS Nonradiation risk factors for thyroid cancer in the US radiologic technologists study Am J Epidemiol 2010 171 2 242 252 10.1093/aje/kwp354 19951937 PMC3290908

[B3] Wiseman S Baliski C Irvine R Anderson D Wilkins G Filipenko D Hemithyroidectomy: the optimal initial surgical approach for individuals undergoing surgery for a cytological diagnosis of follicular neoplasm Ann Surg Oncol 2006 13 3 425 432 10.1245/ASO.2006.03.089 16485160

[B4] Griffith OL Melck A Jones SJM Wiseman SM Meta-analysis and meta-review of thyroid cancer gene expression profiling studies identifies important diagnostic biomarkers J Clin Oncol 2006 24 31 5043 5051 10.1200/JCO.2006.06.7330 17075124

[B5] Jarzab B Wiench M Fujarewicz K Simek K Jarzab M Oczko-Wojciechowska M Gene expression profile of papillary thyroid cancer: sources of variability and diagnostic implications Cancer Res 2005 65 4 1587 1597 10.1158/0008-5472.CAN-04-3078 15735049

[B6] Baris O Mirebeau-Prunier D Savagner F Rodien P Ballester B Loriod B Gene profiling reveals specific oncogenic mechanisms and signaling pathways in oncocytic and papillary thyroid carcinoma Oncogene 2005 24 25 4155 4161 15806164 10.1038/sj.onc.1208578

[B7] Niedzwiecki S Stepien T Kopec K Kuzdak K Komorowski J Krupinski R Angiopoietin 1 (Ang-1), angiopoietin 2 (Ang-2) and Tie-2 (a receptor tyrosine kinase) concentrations in peripheral blood of patients with thyroid cancers Cytokine 2006 36 36 291 295 17374490 10.1016/j.cyto.2007.02.008

[B8] Lin SY Wang YY Sheu WH-H Preoperative plasma concentrations of vascular endothelial growth factor and matrix metalloproteinase 9 are associated with stage progression in papillary thyroid cancer Clin Endocrinol 2003 58 4 513 518 10.1046/j.1365-2265.2003.01749.x 12641636

[B9] Kashat L So AK-C Masui O Wang XS Cao J Meng X Secretome-based identification and characterization of potential biomarkers in thyroid cancer J Proteome Res 2010 9 11 5757 5769 10.1021/pr100529t 20873772

[B10] Johansen JS Lottenburger T Nielsen HJ Jensen JEB Svendsen MN Kollerup G Diurnal, weekly, and long-time variation in serum concentrations of YKL-40 in healthy subjects Cancer Epidemiol Biomarkers Prev 2008 17 10 2603 2608 10.1158/1055-9965.EPI-07-2766 18843001

[B11] Saussez SGD Chantrain G Pattou F Carnaille B André S Gabius HJ Laurent G Serum galectin-1 and galectin-3 levels in benign and malignant nodular thyroid disease Thyroid 2008 18 7 705 712 10.1089/thy.2007.0361 18630998

[B12] Uenishi T Kubo S Hirohashi K Tanaka H Shuto T Yamamoto T Cytokeratin-19 fragments in serum (CYFRA 21-1) as a marker in primary liver cancer Br J Cancer 2003 88 12 1894 1899 10.1038/sj.bjc.6601026 12799633 PMC2741125

[B13] Lukasz A Hellpap J Horn R Kielstein J David S Haller H Circulating angiopoietin-1 and angiopoietin-2 in critically ill patients: development and clinical application of two new immunoassays Critical Care 2008 12 4 R94 10.1186/cc6966 18664247 PMC2575578

[B14] Noji Y Kajinami K Kawashiri M Todo Y Horita T Nohara A Circulating matrix metalloproteinases and their inhibitors in premature coronary atherosclerosis Clin Chem Lab Med 2001 39 5 380 384 11434385 10.1515/CCLM.2001.060

[B15] Breiman L Random forests 2001 Machine Learning: Springer Netherlands 5 32

[B16] Kundel AZR Kato M Moo TA Zhu B Scognamiglio T Fahey TJ 3rd Comparison of microarray analysis of fine needle aspirates and tissue specimen in thyroid nodule diagnosis Diagn Mol Pathol 2010 19 1 9 14 10.1097/PDM.0b013e3181ae870c 20186006

[B17] Gharib H Goellner JR Fine-needle aspiration biopsy of the thyroid: an appraisal Ann Intern Med 1993 118 4 282 289 8420446 10.7326/0003-4819-118-4-199302150-00007

[B18] Huang Y Prasad M Lemon WJ Hampel H Wright FA Kornacker K Gene expression in papillary thyroid carcinoma reveals highly consistent profiles Proc Natl Acad Sci USA 2001 98 26 15044 15049 10.1073/pnas.251547398 11752453 PMC64980

[B19] Bommarito A Richiusa P Carissimi E Pizzolanti G Rodolico V Zito G BRAFV600E mutation, TIMP-1 up-regulation and NF-KB activation: closing the loop on the papillary thyroid cancer trilogy Endocr Relat Cancer 2011 18 6 669 685 10.1530/ERC-11-0076 21903858

[B20] Maeta H Ohgi S Terada T Protein expression of matrix metalloproteinases 2 and 9 and tissue inhibitors of metalloproteinase 1 and 2 in papillary thyroid carcinomas Virchows Archiv 2001 438 2 121 128 10.1007/s004280000286 11253113

[B21] Korem S Resnick MB Kraiem Z Similar and divergent patterns in the regulation of matrix metalloproteinase-1 (MMP-1) and tissue inhibitor of MMP-1 gene expression in benign and malignant human thyroid cells J Clin Endocrinol Metab 1999 84 9 3322 3327 10.1210/jc.84.9.3322 10487706

[B22] Shi Y Parhar RS Zou M Hammami MM Akhtar M Lum Z-P Tissue inhibitor of metalloproteinases-1 (TIMP-1) mRNA is elevated in advanced stages of thyroid carcinoma Br J Cancer 1999 79 7-8 1234 1239 10098765 10.1038/sj.bjc.6690198 PMC2362222

[B23] Kraiem ZKS Matrix metalloproteinases and the thyroid Thyroid 2000 10 1061 1069 10.1089/thy.2000.10.1061 11201850

[B24] Komorowski JPZ Jankiewicz-Wika J Stepien H Matrix metalloproteinases, tissue inhibitors of matrix metalloproteinases and angiogenic cytokines in peripheral blood of patients with thyroid cancer Thyroid 2000 12 655 662 12225633 10.1089/105072502760258622

[B25] Patel A Straight A Mann H Duffy E Fenton C Dinauer C Matrix metalloproteinase (MMP) expression by differentiated thyroid carcinoma of children and adolescents J Endocrinol Invest 2002 25 5 403 408 12035934 10.1007/BF03344028

[B26] Pan S Chen R Crispin DA May D Stevens T McIntosh MW Protein alterations associated with pancreatic cancer and chronic pancreatitis found in human plasma using global quantitative proteomics profiling J. Proteome Res 2011 10 5 2359 2376 10.1021/pr101148r 21443201 PMC3090497

[B27] Lipton A Leitzel K Chaudri-Ross HA Evans DB Ali SM Demers L Serum TIMP-1 and response to the aromatase inhibitor letrozole versus tamoxifen in metastatic breast cancer J Clin Oncol 2008 26 16 2653 2658 10.1200/JCO.2007.15.4336 18443351

[B28] Shao R Hamel K Petersen L Cao QJ Arenas RB Bigelow C YKL-40, a secreted glycoprotein, promotes tumor angiogenesis Oncogene 2009 28 50 4456 4468 10.1038/onc.2009.292 19767768 PMC2795793

[B29] Coffman FD Chitinase 3-Like-1 (CHI3L1): A putative disease marker at the interface of proteomics and glycomics Crit Rev Clin Lab Sci 2008 45 6 531 562 10.1080/10408360802334743 19003601

[B30] Xu XC N-NA Lotan R Differential expression of galectin 1 and galectin-3 in thyroid tumors: potential diagnostic implications Am J Pathol 1995 147 3 815 822 7677193 PMC1870977

[B31] Bartolazzi A Gasbarri A Papotti M Bussolati G Lucante T Khan A Application of an immunodiagnostic method for improving preoperative diagnosis of nodular thyroid lesions The Lancet 2001 357 9269 1644 1650 10.1016/S0140-6736(00)04817-0 11425367

[B32] Türköz HK OH Yurdakul Z Ozcan D Galectin-3 expression in tumor progression and metastasis of papillary thyroid carcinoma Endocr Pathol 2008 19 2 92 96 10.1007/s12022-008-9033-3 18581271

[B33] Htwe TTKN Wong J Jahanfar S Mansur MA Differential expression of galectin-3 in advancing thyroid cancer cells: a clue toward understanding tumour progression and metastasis Singapore Med J 2010 51 11 856 859 21140111

[B34] Prasad ML Pellegata NS Huang Y Nagaraja HN Chapelle A Kloos RT Galectin-3, fibronectin-1, CITED-1, HBME1 and cytokeratin-19 immunohistochemistry is useful for the differential diagnosis of thyroid tumors Mod Pathol 2005 18 1 48 57 10.1038/modpathol.3800235 15272279

[B35] De Matos PS Ferreira AP De Oliveira Facuri F AssumpÃ§Ã£o LVM Metze K Ward LS Usefulness of HBME-1, cytokeratin 19 and galectin-3 immunostaining in the diagnosis of thyroid malignancy Histopathol 2005 47 4 391 401 10.1111/j.1365-2559.2005.02221.x 16178894

[B36] Park MIKD Usefulness of galectin-3, cytokeratin 19, p53, and Ki-67 for the differential diagnosis of thyroid tumors Korean J Pathol 2006 40 86 92

[B37] Raphael S McKeown-Eyssen G Asa S High-molecular-weight cytokeratin and cytokeratin-19 in the diagnosis of thyroid tumors Mod Pathol 1994 7 3 295 300 7520169

[B38] Miettinen M Kovatich AJ Karkkainen P Keratin subsets in papillary and follicular thyroid lesions Virchows Archiv 1997 431 6 407 413 10.1007/s004280050117 9428928

[B39] Sahoo S Hoda SA Rosai J DeLellis RA Cytokeratin 19 immunoreactivity in the diagnosis of papillary thyroid carcinoma Am J Clin Pathol 2001 116 5 696 702 10.1309/6D9D-7JCM-X4T5-NNJY 11710686

[B40] de Araujo VC de Sousa SO Carvalho YR de Araujo NS Application of immunohistochemistry to the diagnosis of salivary gland tumors Appl Immunohistochem Mol Morphol 2000 8 3 195 202 10.1097/00022744-200009000-00005 10981871

[B41] Park YJ KS Kim DC Kim H Choe G Park do J Jang HC Park SH Cho BY Park SY Diagnostic value of galectin-3, HBME-1, cytokeratin 19, high molecular weight cytokeratin, cyclin D1 and p27(kip1) in the differential diagnosis of thyroid nodules Korean Medical Sciences 2007 22 4 621 628 10.3346/jkms.2007.22.4.621 PMC2693809 17728499

[B42] Asahara DCT Takahashi T Fujikawa K Kearney M Magner M Yancopoulos GD Isner JM Tie2 receptor ligands, angiopoietin-1 and angiopoietin-2, modulate VEGF-induced postnatal neovascularization Circ Res 1998 83 233 240 10.1161/01.RES.83.3.233 9710115

[B43] TI Koblizek CW Yancopoulos GD Deutsch U Risau W Angiopoietin-1 induces sprouting angiogenesis *in vitro* Curr Biology 1998 8 529 532 10.1016/S0960-9822(98)70205-2 9560344

[B44] Kanda S Miyata Y Mochizuki Y Matsuyama T Kanetake H Angiopoietin 1 is mitogenic for cultured endothelial cells Cancer Res 2005 65 15 6820 6827 10.1158/0008-5472.CAN-05-0522 16061664

[B45] Thurston G Suri C Smith K McClain J Sato TN Yancopoulos GD Leakage-resistant blood vessels in mice transgenically overexpressing angiopoietin-1 Science 1999 286 5449 2511 2514 10.1126/science.286.5449.2511 10617467

[B46] Witzenbichler B Maisonpierre PC Jones P Yancopoulos GD Isner JM Chemotactic Properties of angiopoietin-1 and -2, ligands for the endothelial-specific receptor tyrosine kinase Tie2 J Biol Chem 1998 273 29 18514 18521 10.1074/jbc.273.29.18514 9660821

[B47] Park JH Park KJ Kim YS Sheen SS Lee KS Lee HN Serum angiopoietin-2 as a clinical marker for lung cancer Chest 2007 132 1 200 206 10.1378/chest.06-2915 17505039

[B48] Yalcin M Dyskin E Lansing L Bharali DJ Mousa SS Bridoux A Tetraiodothyroacetic acid (Tetrac) and nanoparticulate Tetrac arrest growth of medullary carcinoma of the thyroid J Clin Endocrinol Metab 2010 95 4 1972 1980 10.1210/jc.2009-1926 20133461

[B49] Wray CJ Rilo HL Ahmad SA Colon cancer angiogenesis and antiangiogenic therapy Expert Op Invest Drugs 2004 13 6 631 641 10.1517/13543784.13.6.631 15174949

[B50] Hsueh C Lin J-D Wu I-C Chao T-C Yu J-S Liou M-J Vascular endothelial growth factors and angiopoietins in presentations and prognosis of papillary thyroid carcinoma J Surg Oncol 2011 103 5 395 399 10.1002/jso.21844 21400522

